# Spironolactone Lowers Portal Hypertension by Inhibiting Liver Fibrosis, ROCK-2 Activity and Activating NO/PKG Pathway in the Bile-Duct-Ligated Rat

**DOI:** 10.1371/journal.pone.0034230

**Published:** 2012-03-30

**Authors:** Wei Luo, Ying Meng, Hong-Li Ji, Chun-Qiu Pan, Shan Huang, Chang-Hui Yu, Li-Ming Xiao, Kai Cui, Shu-Yuan Ni, Zhen-Shu Zhang, Xu Li

**Affiliations:** 1 Guangdong Provincial Key Laboratory of Gastroenterology, Department of Gastroenterology, Southern Medical University, Nanfang Hospital, Guangzhou, China; 2 Department of Respiratory Diseases, Southern Medical University, Nanfang Hospital, Guangzhou, China; 3 Department of Oncology, 153rd Hospital of People's Liberation Army, Zhengzhou, China; 4 Department of Emergency, Southern Medical University, Nanfang Hospital, Guangzhou, China; 5 Department of Cardiovascular, Southern Medical University, Nanfang Hospital, Guangzhou, China; The University of Hong Kong, Hong Kong

## Abstract

**Objective:**

Aldosterone, one of the main peptides in renin angiotensin aldosterone system (RAAS), has been suggested to mediate liver fibrosis and portal hypertension. Spironolactone, an aldosterone antagonist, has beneficial effect on hyperdynamic circulation in clinical practice. However, the mechanisms remain unclear. The present study aimed to investigate the role of spionolactone on liver cirrhosis and portal hypertension.

**Methods:**

Liver cirrhosis was induced by bile duct ligation (BDL). Spironolactone was administered orally (20 mg/kg/d) after bile duct ligation was performed. Liver fibrosis was assessed by histology, Masson's trichrome staining, and the measurement of hydroxyproline and type I collagen content. The activation of HSC was determined by analysis of alpha smooth muscle actin (α-SMA) expression. Protein expressions and protein phosphorylation were determined by immunohistochemical staining and Western blot analysis, Messenger RNA levels by quantitative real time polymerase chain reaction (Q-PCR). Portal pressure and intrahepatic resistance were examined in vivo.

**Results:**

Treatment with spironolactone significantly lowered portal pressure. This was associated with attenuation of liver fibrosis, intrahepatic resistance and inhibition of HSC activation. In BDL rat liver, spironolactone suppressed up-regulation of proinflammatory cytokines (TNFα and IL-6). Additionally, spironolactone significantly decreased ROCK-2 activity without affecting expression of RhoA and Ras. Moreover, spironolactone markedly increased the levels of endothelial nitric oxide synthase (eNOS), phosphorylated eNOS and the activity of NO effector- protein kinase G (PKG) in the liver.

**Conclusion:**

Spironolactone lowers portal hypertension by improvement of liver fibrosis and inhibition of intrahepatic vasoconstriction via down-regulating ROCK-2 activity and activating NO/PKG pathway. Thus, early spironolactone therapy might be the optional therapy in cirrhosis and portal hypertension.

## Introduction

In cirrhosis, increased intrahepatic resistance is the primary event causing portal hypertension [1]–[3]. Both intrahepatic fibrosis and imbalance between vasoconstrictor and vasodilator mediators contribute to increased resistance [4]–[5]. In these circumstances, activated hepatic stellate cells (HSCs) play a key role via transdifferentiation to myofibroblasts-like to acquire contractility and result in extracellular matrix (ECM) deposition [4]–[5].

Aldosterone, one of the main peptides in the RAAS, has been suggested to mediate inflammation, oxidative stress, endothelial dysfunction and fibrosis [6]–[7]. Existing studies of aldosterone inhibitors have showed that the mineralocorticoid receptor (MR) antagonist reduces fibrogenesis and lowers portal hypertension [8]–[9]. However, the molecular mechanisms by which spironolactone induces these effects remain unclear.

It is well known that in cirrhosis activated RhoA/ROCK-2 signaling and inhibited nitric oxide (NO) availability contribute to increased intrahepatic resistance and portal hypertension [5]. Increased RhoA/ROCK-2 reduces the NO synthase activity via down-regulating the levels of endothelial nitric oxide synthase (eNOS). NO, in turn, induces vasorelaxation through the activation of cyclic guanosine 3′, 5′-monophosphate (cGMP)/protein kinase G (PKG) [10].

Furthermore, our recent in vitro finding showed that aldosterone induced contraction of activated HSCs by activation of the RhoA/ROCK-2 signaling pathway, while spironolacton and the ROCK-2 inhibitor Y27632 could suppress this effect [11]. Therefore, the aim of the present study was to investigate the effect of chronic spironolactone treatment on intrahepatic RhoA/ROCK-2 signaling and NO/PKG pathway as well as on lilver fibrosis and portal hypertension.

## Materials and Methods

### Animal

Male Wistar rats weighing 200–300 g were purchased from the Laboratory Animal Center (Southern Medical University, China). All experimental procedures on rats were approved by the Committee on the Ethics of Animal Experiments of Southern Medical University (Permission No.: 2009-015). Animals were housed under a controlled environment (12 hours light/12 hours dark; temperature, 22–24°C), and received water *ad libitum* in the Animal Care Facility Service (Southern Medical University, China). All surgery was performed under Phenobarbital sodium anesthesia, and all efforts were made to minimize suffering. This study was carried out in strict accordance with the recommendations in the Guide for the Care and Use of Laboratory Animals of the National Institutes of Health.

### Treatment regimens

Billary hepatic fibrosis was induced by double ligation and transection of the common bile duct, as previously described [12], [13]. Spironolactone or vehicle (saline) was administered orally by gavage. Eighteen rats underwent BDL and sacrificed at two weeks (n = 8) and 4 weeks (n = 10). The rats in BDL+spironolactone treatment group were administered with sprionolactone (20 mg/kg body weight per day) once a day after bile duct ligation and sacrificed at 2 weeks (n = 8) and 4 weeks (n = 10). This dosage was chosen according to the literature [14]. Sham-operated rats (n = 10) served as controls. In these rats, the common bile duct was exposed by only median laparotomy, neither ligation nor resection was performed.

### Tissue collection and biochemical analyses

After the indicated periods, blood was obtained for the measurement of biochemical parameters (AST, ALT, and bilirubin) using standard methods. The liver was cut into fragments. Liver samples were either stored in formaldehyde or snap-frozen in liquid nitrogen and stored at −80°C as previously described [15], [16].

### Histological and immunochemical assessment

Sections of liver (4 µm) mounted on silane-coated glass slides were stained with haematoxylin and eosin (H&E), immunohistochemical and Masson's trichrome collagen staining. Liver sections were assessed in random order by an experienced liver pathologist, who was blinded to the animal groups. Sections were assessed for METAVIR fibrosis score and the ductal proliferation score, as adapted from Miyoshi et al. [17]. The number of biliary infarcts was also documented for each field examined.

For immunohistochemistry, the sections were incubated with primary antibody (Cell Signaling Technology, Danvers, MA) in concentrations of 1∶200 (phosphor-Thr558-moesin), 1∶200 (phospho-Ser239-VASP) and (Abcam plc, Cambridge, UK) 1∶100 (α-SMA), followed by incubation with streptavidin–peroxidase complex. Peroxidase conjugates were subsequently visualized by utilizing diaminobenzidine (DAB) solution. The sections were then counterstained with hematoxylin and mounted on a cover slip.

Masson's trichrome collagen staining was quantified for collagen by analyzing Masson-stained area as a percentage of total area. We averaged the values of the sections from three rats in each group.

### Hepatic hydroxyproline determination

Collagen content of the liver was quantified using hydroxyproline detection kit (Jiancheng Institute of Biotechnology, Nanjing, China) according to the manufacturer' s instructions. All experiments were performed in triplicates. Results are expressed as ug/g of wet liver tissue.

### Western Blotting

Western blotting was performed as described previously [11]. The primary antibodies were α-SMA (Abcam plc, Cambridge, UK), type I collagen (Sigma–Aldrich Corporation, Saint Louis, MO, USA), Ras, Rhoa, moesin, p-moesin, vasodilator-stimulated phosphoprotein [VASP], p-moesin (Cell Signaling Technology, Danvers, MA) or GAPDH (Beijing, Zhongshan Biotech Co, China). For protein quantification, bands were scanned and quantified with GAPDH as an internal control. Western blot analyses from all groups were calibrated to sham-operated rats set to 100 densitometric units (d.u.).

### Quantitative PCR

RNA was isolated from 30 mg of liver tissue following the manufacturer's protocols of Trizol isolation (TaKaRa Bio, Japan). RNA (2 µg) was reverse-transcribed using PrimeScript™ RT reagent kit (00057250, Fermentas, EU), and the single- stranded cDNA was amplified by quantitative real-time RT-PCR using SYBR green Master Mix kit (04913914001, Roche, USA) on an ABI PRISM 7500 TNFα, (Forward) 5′-CGT CGT AGC AAA CCA CCA AG-3′ and (Reverse) 5′- CAC AGA GCA ATG ACT CCA AAG-3′; IL-6, (Forward) 5′-CCA CTG CCT TCC CTA CTT-3′ and (Reverse) 5′- TTG GTC CTT AGC CAC TCC-3′; CYP11B2 (aldosterone synthase gene), (Forward) 5′- TGG CTG AAG ATG ATA CAG ATC CT-3′ and (Reverse) 5′- CAC TGT GCC TGA AAA TGG GC-3′; RhoA GTP, (Forward) 5′-CAG CAA GGA CCA GTT CCC AGA-3′ and (Reverse) 5′-AGC TGT GTC CCA TAA AGC CAA CTC-3′; Rho GEFs, (Forward) 5′-TGC CCA ACC AGG AGC AAT C-3′ and (Reverse) 5′-TGC AAT CTC AAG CAC CTG GAA-3′; ROCK-2, (Forward) 5′-CTA ACA GTC CGT GGG TGG TTC A-3′ and (Reverse) 5′-TCC ACC TGG CAT GTA CTC CAT C-3′; eNOS, (Forward) 5′-CTA CCG GGA CGA GGT ACT GG-3′ and (Reverse) 5′-GGA AAA GGC GGT GAG GAC TT-3′ and GAPDH, (Forward) 5′-GGC ACA GTC AAG GCT GAG AAT G-3′ and (Reverse) 5′-ATG GTG GTG AAG ACG CCA GTA-3′. The cycles for PCR were as follows: one cycle of 95°C for 10 minutes, 40 cycles of 15 seconds at 95°C, 1 minute at 60°C and a final 1 minute at 60°C. The mRNA expression of the target gene was normalized to GAPDH.

### Assessment of PKG and ROCK-2 activity

PKG activity was assessed as phosphorylation of the endogenous PKG substrate, VASP, at Ser-239. The phosphorylation state of VASP served as a marker for PKG activity [18]. ROCK-2 activity was assessed as phosphorylation of the endogenous Rho-kinase substrate, moesin, at Thr- 558 [19], [20]. This was done by Western blot analysis using site- and phospho-specific antibodies.

### Measurement of portal pressure

Rats in all groups were fasted for 12 hours and anesthetized with an intraperitoneal injection of Phenobarbital sodium (50 mg/kg bodyweight). Portal flow (F) was calculated by color Doppler flow imaging (CDFI, Acuson Seguoia 512, Simens) according to the measured the inner diameter (D) and maximized blood flow velocity (V): (F = 0. 57π*D*2 *V*/4×60). Subsequently, median laparotomy was performed, and a PE-50 catheter was inserted into the ileocolic vein and advanced to the portal vein. The cannula was used for the measurement of portal pressure (PP) via connecting to a pressure transducer (Power laboratory, AD Instruments, Australia).

### In situ liver perfusion

In situ liver perfusion was performed as previously described [21]. Briefly, after anesthesia with Phenobarbital sodium (50 mg/kg bodyweight. i.p), the abdomen of rat was opened, and the bile duct was cannulated with a polyethylene tube to monitor bile flow. Loose ligatures were placed around the inferior vena cava (IVC) above the right renal vein. The portal vein was cannulated with a 14-gauge Teflon catheter, and the liver was perfused with Krebs-Henseleit solution (pH 7.4, 37°C) at a constant flow rate. The perfusion buffer contained heparin (2 IU/ml) and was oxygenated with carbogen (95%O_2_, 5%CO_2_). Subsequently, the abdominal aorta and IVC were cut caudally to the loose ligature, allowing the perfusate to escape. Then the IVC was cannulated via the right atrium and ligated immediately. Portal perfusion pressure (PPP) was monitored continuously. Through pressure transducers the results were transmitted to a Powerlab/4sp-linked computer using Chart version 4.0 for Windows (AD Instruments).

The viability of each liver was assessed by gross appearance, stable perfusion and bile production. If any of the criteria were not satisfied, the sample was discarded.

### Statistics analysis

Data were summarized as mean ± standard error of the mean (S.E.M.) based on experiments repeated in triplicate. Multiple comparisons were analyzed using one-way analysis of variance (ANOVA) with Statistical Package for the Social Sciences (SPSS) 13.0 software (Chicago, IL). A probability (*p*)-values less than 0.05 were considered statistically significant.

## Results

### Effect of spironlactone on liver fibrosis

**Figure 1:**
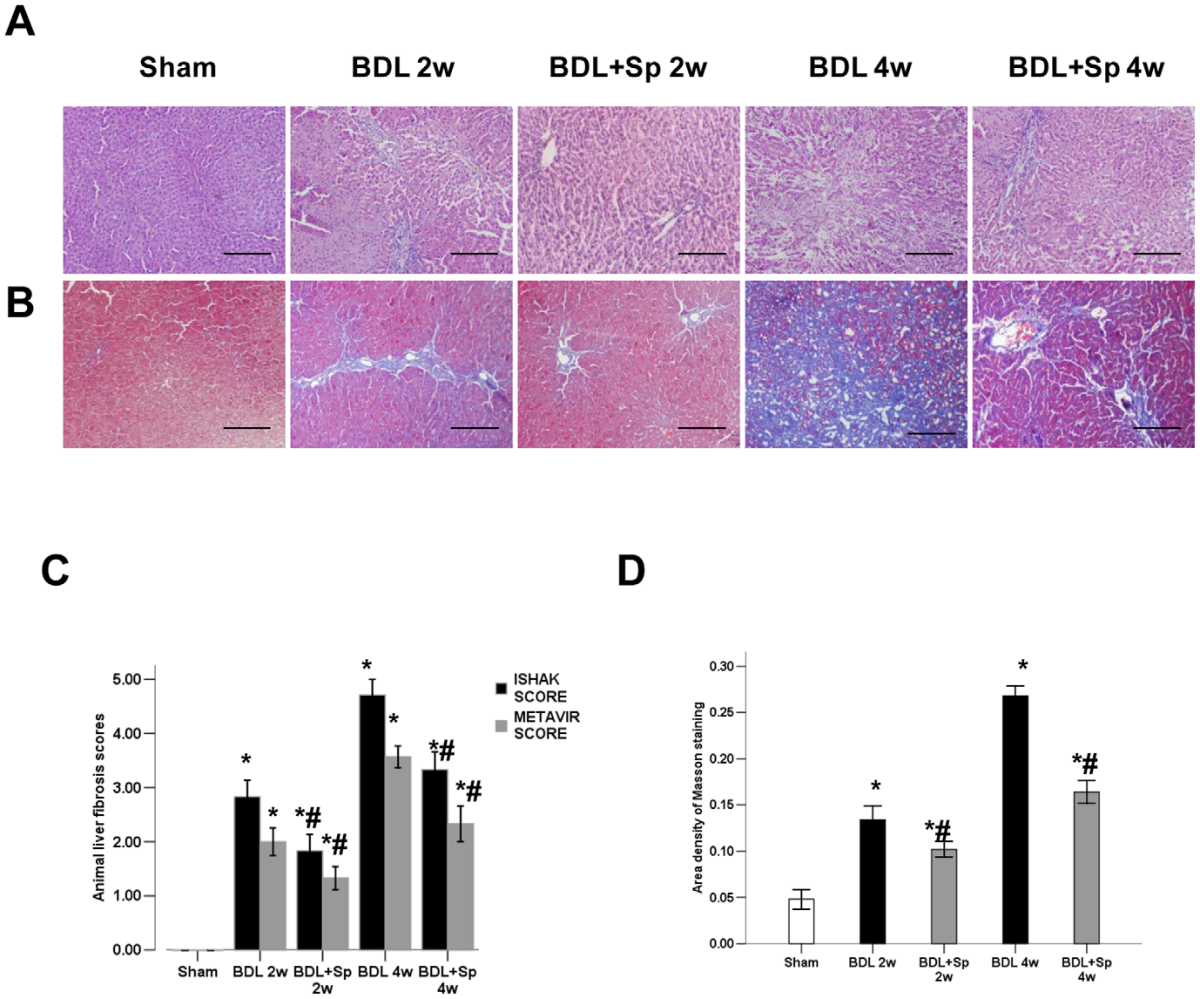
Therapeutic effects of Spironolactone (Sp) on hepatic fibrosis in BDL rats. Histological images of rat livers stained with H&E (A) or Masson (B) (magnification 200×). Liver fibrosis scores (C) and Semiquantitative measurement of Masson staining (D) in spironolactone or vehicle-treated BDL rats. *p<0.05 compared to the Sham group. #*p*<0.05 compared to the BDL groups.

**Figure 2:**
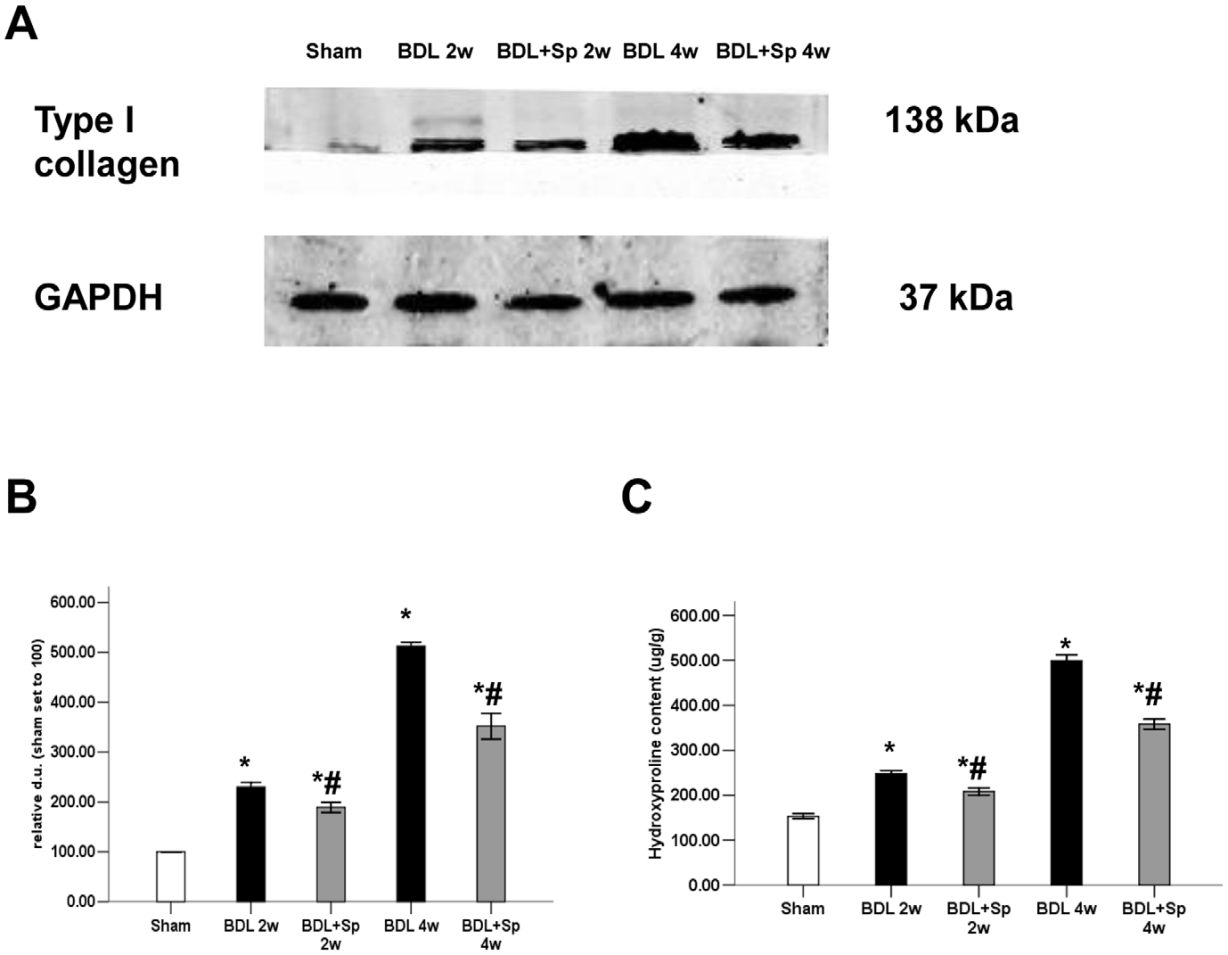
Spironolactone (Sp) downregulates type I collagen expression and reduces hydroxyproline content in BDL rats. (A) Spironolactone downregulates type I collagen protein expression in the livers of BDL rats, as shown by Western blot analysis (B). Sprionolactone reduces hydroxyproline content in rat livers (C). *p<0.05 compared to the Sham group. #*p*<0.05 compared to the BDL groups.

BDL caused significant histological changes, including a distortion of the normal architecture, expansion of portal tracts with extensive bile-duct proliferation and deposition of collagen (Figure 1A). Spironlactone attenuated liver fibrosis and decreased the collagen deposition significantly compared with the BDL group during two and four weeks. Hepatic hydroxyproline content was increased in BDL-treated rats, while treatment with spironlactone significantly inhibited the secretion of hydroxyproline by 11.2% (two weeks) and 31.3% (four weeks) respectively (Figure 2C). This could be confirmed histologically using Masson's staining (Figure 1B) and the expression of type I collagen by Western blot analysis (Figure 2B). In addition, there was a significant increase in the analyzed biochemical parameters (bilirubin, ALT, and AST) in BDL rats as compared to sham-operated animals (Table 1).

**Table 1:** Biochemical parameters of different groups (mean ± SD).

Group	ALT (U/L)	AST (U/L)	BIL (Ul/dL)
Sham	4.08±6.54	24.09±5.24	15.34±3.26
BDL 2w	53.27±2.56*	60.75±2.54*	31.98±3.65*
BDL+Spironolactone 2w	58.78±2.65*	57.33±7.43*	28.54±4.32*
BDL 4w	73.42±8.25*	68.34±6.37*	38.36±4.25*
BDL+Spironolactone 4w	67.09±6.76*	73.34±5.29*	34.04±3.32*

* *p*<0.05 compared to the Sham group.

### Spironlactone reduced HSCs accumulation

**Figure 3:**
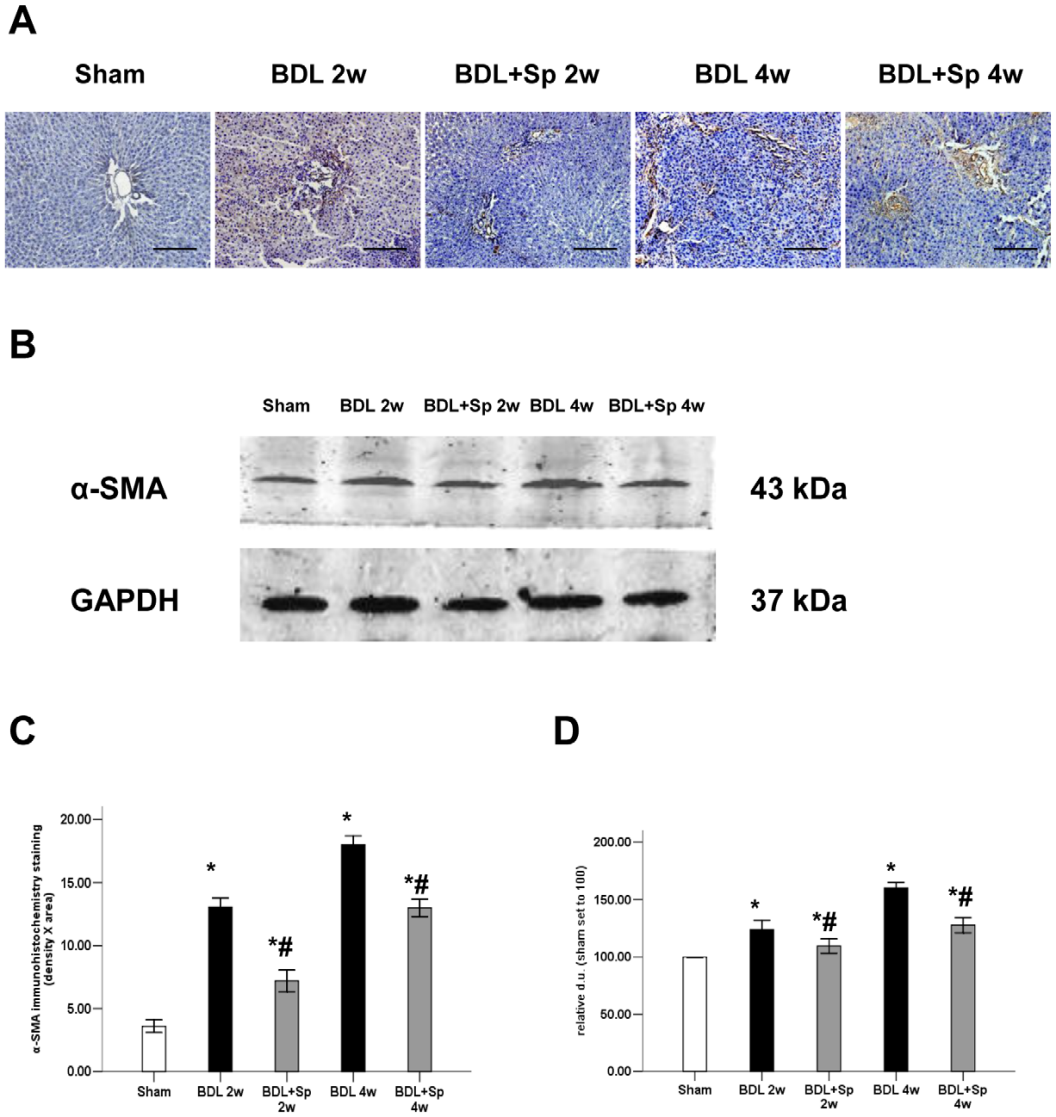
Effects of therapeutic treatment with Sprionolactone (Sp) on hepatic HSCs accumulation, assessed by (A) immunohistochemistry for hepatic α-smooth muscle actin (α-SMA) (magnification 200×) and (B) Western blot analysis of α-SMA expression. Semiquantitative measurement of immunohistochemistry (C) and Western blot analysis (D) for α-SMA. *p<0.05 compared to the Sham group. #*p*<0.05 compared to the BDL groups.

To evaluate the effect of spironolacton treatment on activity of HSCs, immunohistochmical staining for α-SMA was performed (Figure 3A). Increased α-SMA staining was observed in livers of BDL rats during weeks 2 and 4 of the experiment. To compare with the BDL group, there was a reduction in the number of α-SMA positive cells in the spironolacton treatment group. These findings were substantiated by Western blot analysis which showed that spironlactone treatment significantly reduced hepatic α-SMA expression (Figure 3B).

### Effect of spironolactone on the inflammatory genes and aldosterone synthase gene expressions

**Figure 4:**
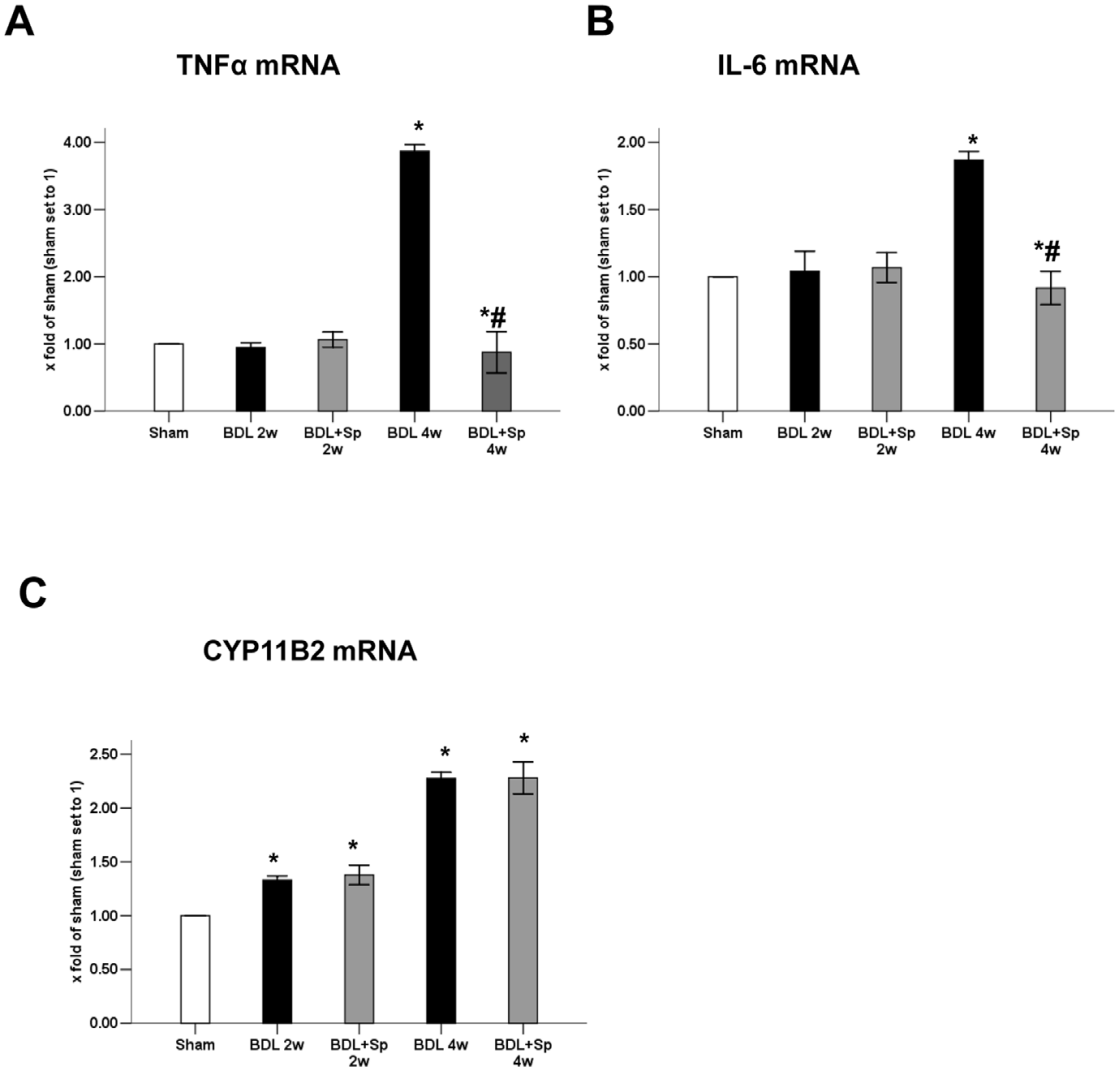
Effects of spironolactone on the inflammatory genes and aldosterone synthase gene expressions in BDL-treated rats. (A) Real-time PCR analysis for TNFαmRNA expression. (B) Real-time PCR analysis for IL-6 mRNA expression. (C) Real-time PCR analysis for CYP11B2 mRNA expression. *p<0.05 compared to the Sham group. #*p*<0.05 compared to the BDL groups.

Since spironolactone is suggested to be an inflammatory inhibitor, it is worthwhile to investigate whether the protective effect is mediated through its anti-inflammatory function. Our present study showed that intrahepatic mRNA expression of TNFα and IL-6 was significantly elevated in BDL rats at four weeks (Fig. 4A, 4B). Notably, spironolactone markedly decreased the expressions of TNFα and IL-6 respectively compared with BDL rats. Whereas at two weeks, neither TNFα nor IL-6 incresed significantly in BDL rats compared to sham rats. In addition, aldosterone is a downstream mediator of RAAS and CYP11B2 is the key synthase of aldosterone. Our results found that CYP11B2 notably up-regulated in BDL groups compared to sham-operated group (Fig. 4C).

### Spironolactone inhibited ROCK-2 activity

**Figure 5:**
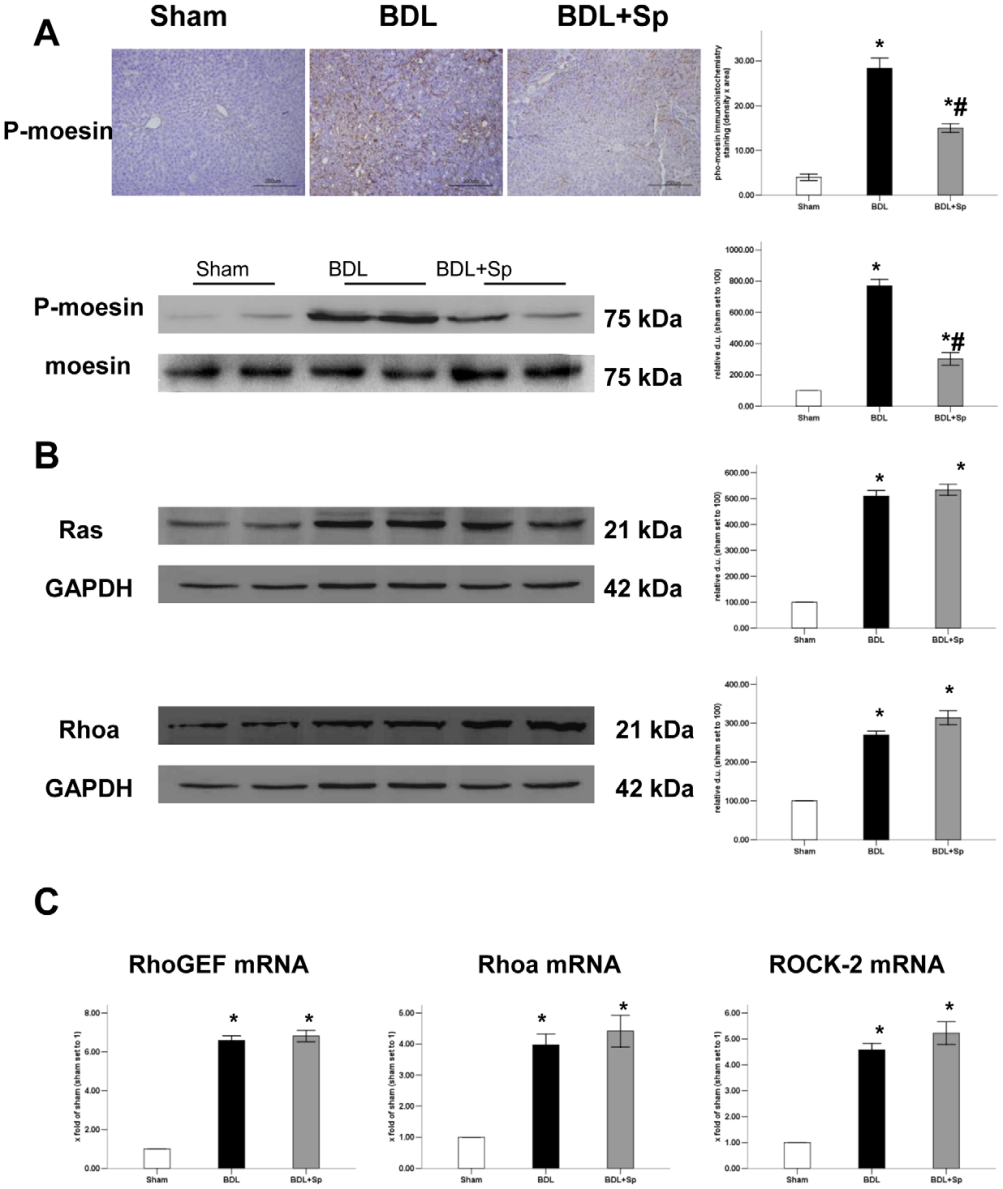
Sprionolactone (Sp) inhibits the phosphorylation of moesin in BDL- treated rats. (A) immunohistochemistry for hepatic phosphor-moesin of paraffin-embedded liver sections (magnification 200×) and Western blot analysis of p-moe expression. (B) Expression of Ras and RhoA proteins in liver homogenates as determined by immunoblot. (C) Real-time PCR analysis for RhoGEF, RhoA and ROCK-2 mRNA expression. *p<0.05 compared to the Sham group. #*p*<0.05 compared to the BDL groups.

Western blot analysis showed that intrahepatic protein levels of RhoA and Ras were increased in BDL rats compared with sham-operated rats. Treatment with spironlactone did not affect the expression of RhoA and Ras proteins (Figure 5B). Similarly, as revealed by Q-PCR with mRNA isolated from whole liver homogenates, RhoA, RhoGEF, ROCK-2 mRNA levels were significantly increased in the BDL rats compared to that in sham-operated rats. In the spironlactone-treated rats, mRNA levels remained unchanged when compared with those of untreated BDL rats (Figure 5C).

As a marker of ROCK-2 activity, phosphorylation of moesin was investigated by immunohistochemical staining and Western blot. Analysis of these stainings showed a reduction in the number of phosphor-moesin positive cells in the spironolactone treatment group compared to BDL group. In addition, Western blot showed that phosphor-moesin greatly increased in the livers of BDL rats. Spironolactone treatment significantly decreased the intrahepatic phosphorylation of moesin. This difference was not associated with changes in total moesin, which was similar in all groups (Figure 5A). Since moesin is phosphorylated at Thr-558 by ROCK-2, these findings probably reflected chronic spironolactone treatment inhibits ROCK-2 activity in the liver of BDL rats.

### Spironolactone increased NO/PKG pathway

**Figure 6:**
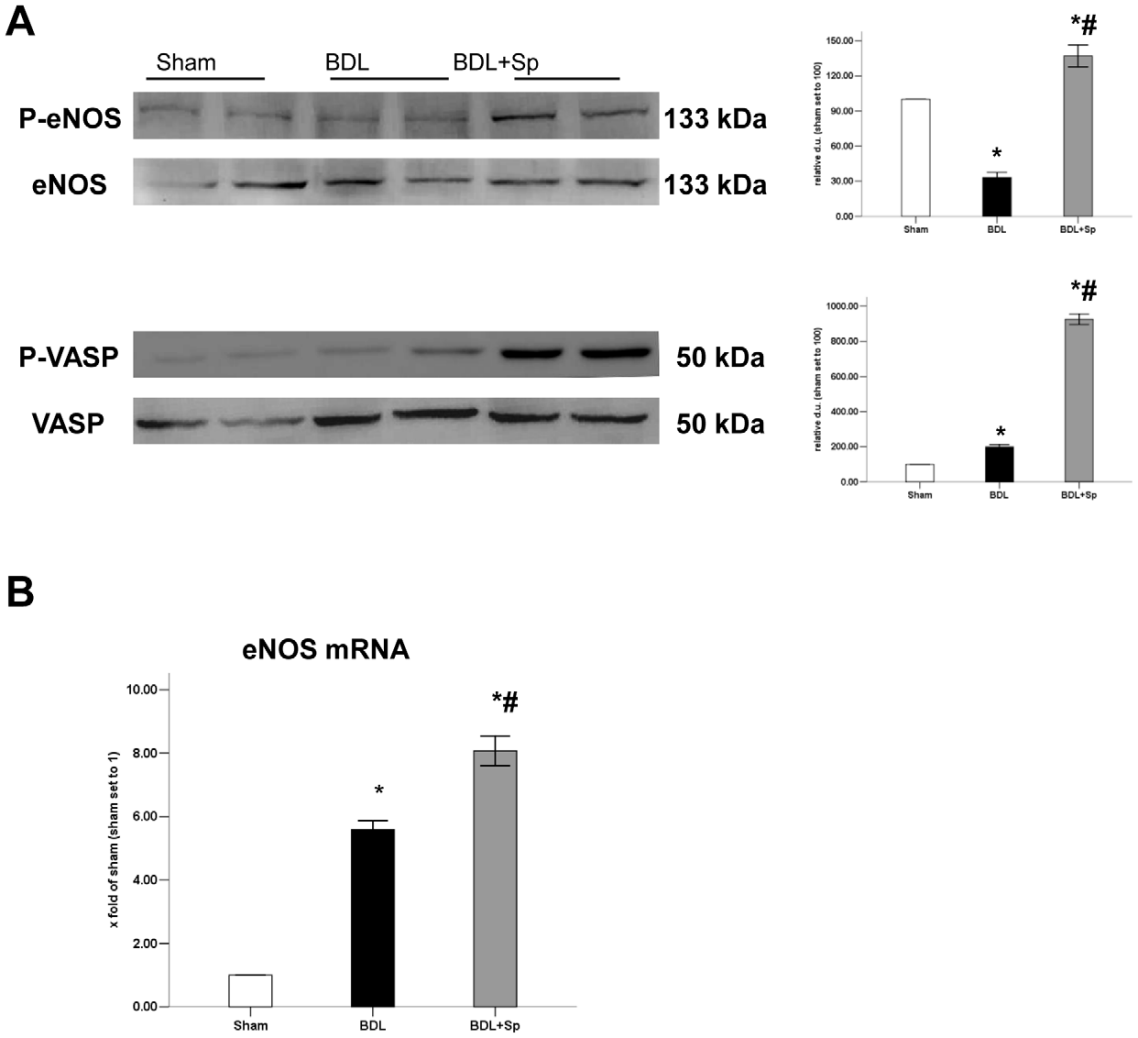
Sprionolactone (Sp) increased the phosphorylation of vasp in BDL- treated rats. (A) Western blot analysis of the total- or phospho-vasp and eNOS in BDL rats. The expression levels of phospho-vasp and eNOS are measured relative to the total-vasp and eNOS respectively. (B) Real-time PCR analysis for eNOS mRNA expression. *p<0.05 compared to the Sham group. #*p*<0.05 compared to the BDL groups.

Western blot analysis revealed that sprionolactne had no effect on eNOS protein levels of the livers, but a decrease in phosphorylation of eNOS was found in BDL rats compared with sham-operated rats. Treatment with spironolactone increased the intrahepatic phospho-eNOS content (Figure 6A). In parallel, mRNA levels of eNOS were greatly increased when compared with that of BDL rats (Figure 6B).

Phosphorylation of VASP, which is phosphorylated at Ser-239 by PKG, was determined as a marker of PKG activity. Western blot analysis showed that phospho-VASP was elevated in the livers of BDL rats compared to sham-operated rats. Spironolactone treatment significantly increased the levels of phosphorylation of VASP. These differences were unrelated to total VASP, which remained unchanged in all three groups. Thus, spironolactone enhances intrahepatic PKG activity in BDL rats (Figure 6A).

### Effect of spironolactone on portal hypertension and intrahepatic resistance

**Figure 7:**
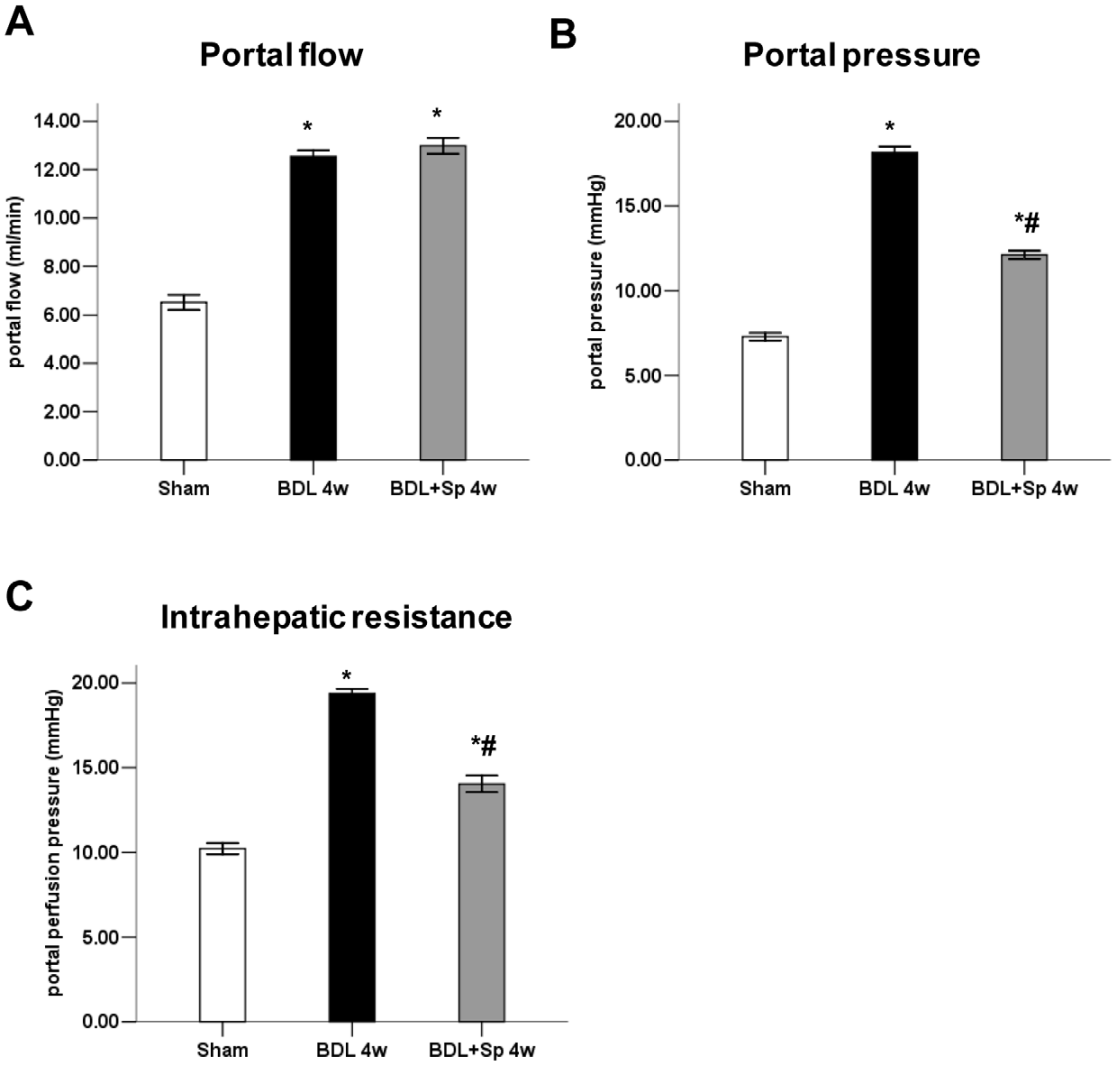
Spironolactone (Sp) improves portal pressure and lower portal vein resistance in BDL- treated rats. (A) Quantification of portal flow in the sham, BDL and BDL+Sp groups. (B) Portal pressure, (C) intrahepatic resistance in situ liver perfusion (20 ml/min) in sham-opreated rats, untreated cirrhotic BDL rats, and cirrhotic BDL rats treated with Sp. *p<0.05 compared to the Sham group. #*p*<0.05 compared to the BDL groups.

As expected, the portal vein flow was significantly elevated in BDL rats compared with that in sham- operated rats. Spironolactone did not affect the portal vein flow in BDL rats. Furthermore, the portal pressure in BDL rats was markedly higher than that of in sham-operated rats. However, spironolactone treatment significantly attenuated increased portal pressure induced by BDL. Similarly, hepatic vascular resistance was increased in cirrhotic rats compared to sham-operated rats and spironolactone administration significantly decreased hepatic resistance (Figure 7). These results suggested that chronic spionolactone treatment ameliorated intrahepatic resistance and portal hypertension.

## Discussion

Spironlacton, an aldosterone antagonist, has been extensively used as a minor diuretic in achieving volume homeostasis. Although sprionolactone treatment maybe effective in patients with cirrhotic ascites and portal hypertension [22], [23], little is known about its potential mechanism. In this study, we found that spironolacton limited the development of liver cirrhosis and lowered portal hypertension. This was associated with inhibition of activated HSCs and reduction intrahepatic resistance in BDL-treated rats. Furthermore, sprionolactone inhibited hepatic RhoA/ROCK-2 and activated hepatic NO/PKG signaling.

In vitro studies demonstrated that aldosterone induced the synthesis of procollagen I and IV in rat HSCs [24]; canrenone, an anti-aldosterone drug, reduced cells proliferation, migration and synthesis of procollagen I and IV in human HSCs [25]. Our present study showed that spironolactone treatment substantially ameliorated the extent of fibrosis in the BDL rat, as assessed by METAVIR and ISHAK fibrosis scoring systems and computerized morphometric quantification of Masson's staining. In addition, we observed that spironolactone markedly decreased the hydroxyproline content, a good marker of ECM accumulation [26], and the expression of type I collagen in the liver of cirrhotic rat. These results suggested that by inhibiting the accumulation of ECM components, spironolactone treatment ameliorated the structural derangements that increased intrahepatic resistance and portal hypertension [27].

To further investigate the involvement of HSCs in this process, we performed immunohistochemical staining for α-SMA, a marker of activated HSCs which play central roles in liver fibrogenesis [28], [29]. The reduction areas of staining for α-SMA indicated a decreased number of activated HSCs after spironolactone treatment. Western blot analyses also confirmed that there was a downregulation of α-SMA expression in spironolactone treated rats. The differences between BDL and BDL+Sp groups were minor though they were statistically significant. Thus the reduced α-SMA content in the experiment suggests that spironolactone decrease the activity status of HSCs with contractile property. This hypothesis was supported by our previous study [11]. As HSCs activation is closely responsible for ECM production, intrahepatic angiogenesis and vascular remodeling in cirrhotic liver [29], [30], this indicates that spironolactone reduces intrahepatic resistance and consequently results in a reduction of portal hypertension via an HSC-dependent manner.

Our previous in vivo study confirmed that aldosterone synthase key gene CYP11B2 was upregulated in CCl_4_-induced cirrhotic rat liver [31]. Tsutomu Wada et al also showed that CYP11B2 elevated in high fat and high fructose diet (HFFD) mice and HFFD+spironolactone mice [32]. Similarly, in our present study, we found that the mRNA expression of CYP11B2 gradually increased with the aggravation of fibrosis in BDL rat liver. These results suggested that mineralocorticoid receptor (MR) antagonist had no impact on the expression of aldosterone synthase.

Increasing evidences demonstrated that aldosterone per se promotes inflammation and reactive oxygen species (ROS) production in vessels [33], [34], kidney [35], [36], heart [37] and liver [32]. However, mineralocorticoid receptor (MR) antagonist could suppress inflammation and ROS production. In addition, our previous in vitro study also found that aldosterone increased HSCs NF-κ B activity and NF-κ B target gene-TNFα expression by inhibiting IκBα expression in a redox-sensitive manner [7]. The present study showed that increased expressions of TNFα and IL-6 in the liver of BDL rat are significantly suppressed in spironolactone treatment group at four weeks (Fig. 4A, 4B). It is possible that spironolactone plays the protective role through its anti-inflammatory function.

According to our previous study, in vitro aldosterone markedly upregulated the active RhoA (RhoA GTP) protein expression in HSCs. The effect was suppressed by both the MR inhibitor spironolactone and the ROCK-2 inhibitor Y27632. Moreover, spironolactone can inhibit activated HSCs contraction induced by aldosterone via RhoA/ROCK-2 signaling pathway [11]. Current studies have demonstrated that RhoA/ROCK-2 pathway is essentially involved in vasoconstriction and the regulation of vascular tone [5], [20]. In our present experiment, there was a strong upregulation of RhoA and Ras protein expression as well as RhoA, Rho GEFs and ROCK-2 mRNA expression in livers of BDL rats. Besides, the hepatic upregulation of RhoA and ROCK-2 resulted in an increased moesin phosphorylation, reflecting an increased activity of ROCK-2. While sprionolactone significantly decreased the levels of phosphor-moesin in immunohistochemical staining and western blot analyses without altering hepatic expression of RhoA and total moesin. This indicates that spironolatcone decreased hepatic ROCK-2 activity. As revealed by our haemodynamic measurements, spironolactone lowered portal pressure and reduced intrahepatic resistance in the in situ perfused liver model. Therefore, combined with our previous in vitro study we speculated that spironolactone might directly reduce activation of cells contraction mediated by ROCK-2 and thus decrease intrahepatic resistance.

As RhoA/ROCK-2 negatively regulate eNOS mRNA stability [38], [39], inhibition of RhoA/ROCK-2 with spironolactone might increase eNOS expression and NO production. In the present study, we first showed that spironolactone upregulatd the expression of eNOS mRNA and protein in the liver of BDL rat, which was accompanied with an increase of phospho-eNOS content (Ser-1177). Meanwhile, spironolactone increased the levels of phospho-VASP, a substrate of PKG, and subsequently mediated NO-induced vasorelaxation. In addition, there is evidence supporting PKG- dependent inactivation of RhoA [40]. Taken together, we assumed that sprionolactone could increase intrahepatic NO production and resulte in vasodilation.

Current innovative treatment methods attempt to attenuate hepatic fibrosis or lower portal hypertension by inhibition HSC survival and growth or by increasing the production and bioavailability of NO [41], [42]–[43], [44]. However, none of these treatment methods is applicable in clinical setting. On the contrary, patients may be easily treated with spironolactone since it is available for pennies a day. In our experimental study, we demonstrated chronic treatment with spironolactone lowered portal pressure by antifibrogenic effect and inhibition of intrahepatic vasoconstriction. These findings warrant further investigation in other cirrhosis models such as CCl_4_ and long-term studies in humans.

In summary, spironolactone was effective in lowering portal hypertension in cirrhotic BDL rats. This is attributed to its antifibrotic activity and decreasing intrahepatic resistance via inhibition of RhoA/ROCK-2 pathway and activation of NO/PKG signaling. This suggests that spironolactone may benefit patients with liver cirrhosis and portal hypertension besides of diuretic effect when perform appropriate monitoring of renal and electrolyte status.
